# Applicator for cervical brachytherapy for MRI or CT guided therapy

**DOI:** 10.1016/j.tipsro.2021.10.002

**Published:** 2021-10-30

**Authors:** Nicola J. Nasser

**Affiliations:** a Department of Radiation Oncology, School of Medicine, Maryland Proton Treatment Center, University of Maryland Baltimore, Baltimore, MD 21201, USA; b The Umbilicus Inc., Nonprofit Organization for Preserving Sexual Function of Individuals with Cancer Below the Umbilicus, New York, NY, USA

**Keywords:** Cervical Cancer, Brachytherapy, Applicator, Radiation

## Abstract

Cervical cancer is the second leading cause of cancer death in women aged 20 to 39 years in the USA. Radiation therapy for cervical cancer in the definitive setting is delivered by combining external beam radiation and brachytherapy. The procedure of cervical brachytherapy could be associated with significant discomfort and pain to the patient. Here we describe a novel cervical applicator, that can be inserted bedside, with local anesthesia only. The device is similar to intrauterine device and vaginal ring used for contraception, albeit with channels for high dose rate brachytherapy.

## Highlights

• Cervical applicator for brachytherapy with anchorage similar to intrauterine device.
• Cervical applicator that can be inserted bedside.
• Cervical applicator that can be associated with less pain compared to current applicators.

## Graphical abstract

**Figure:**
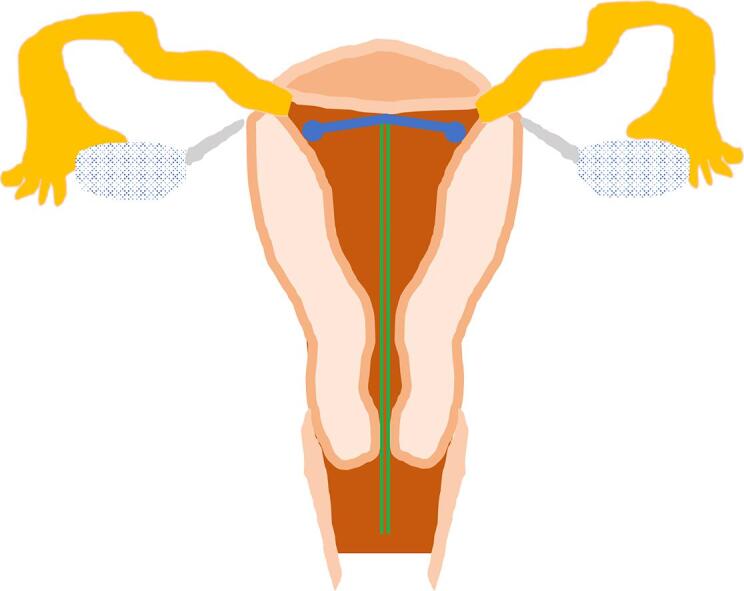

## Introduction

Cervical cancer is the second leading cause of cancer death in women aged 20 to 39 years in the United States of America (USA) [1]. During 2021, 14,480 new cases of cervical cancer are expected to be diagnosed, and 4,290 women are expected to die due to cervical cancer in the USA [1]. Worldwide cervical cancer is the fourth most frequently diagnosed malignancy and the fourth leading cause of cancer death in women, with an estimated 604,000 new cases and 342,000 deaths in 2020 [2].

While very early-stage cervical cancer can be cured with surgery, locally advanced disease needs definitive treatment with chemotherapy and radiation [3], [4]. Radiation therapy for locally advanced cervical cancer in the definitive setting is delivered by combining external beam radiation and brachytherapy [5]. Brachytherapy provides high doses of radiation to the cervix, while sparing nearby organs at risk from high doses of radiation.

Brachytherapy for cervical cancer is a multistep procedure. Classic intracavitary applicators include tandem and ovoids, tandem and ring, and tandem and vaginal cylinder [6]. Interstitial applicators include needles that can be inserted into tumors directly and have a great dose advantage for bulky tumors, lateral parametrial or pelvic wall extension [6]. For intracavitary brachytherapy, the first step, involves cervical dilatation, which is done under general or spinal anesthesia. Serial dilatations are performed starting with a thin, low Hegar number rod and progressing gradually to larger numbers. After that, Smit sleeve is inserted into the cervical canal, and stitched to it, to ensure that the cervix remains patent for the brachytherapy treatment [7]. After the patient recovers and discharged, she comes to 4–5 treatments on different days, during which a tandem is inserted into the Smit sleeve, and ring or ovoids are attached to the tandem at its both sides, to allow delivery of radiation into the ring or ovoids channels, to treat the cervix and tissues immediately near it.

While brachytherapy using current applicators is highly effective, the procedure can be very painful for the patient. Surgical dilatation of the cervix needs an operation room, high expertise in the procedure to avoid rupture of the uterus, and time and experience for stitching the cervical Smit sleeve.

According to the American Brachytherapy Society consensus guidelines for locally advanced carcinoma of the cervix [8], the procedure of insertion of intracavitary applicators is typically serial and a painful or unpleasant experience may inhibit the patient from completing the recommended therapy and decrease the quality of medical care [8]. Different methods are used to control patients’ pain during the insertion of the intracavitary cervical applicator. A survey of gynecologic cancer experts for the type of analgesia administered for cervical cancer brachytherapy, included general (46%), spinal (27%), intravenous conscious sedation (28%), and/or oral pain medication (14%) [9].

The current invention is an applicator for delivery of high dose rate brachytherapy for cervical cancer, that can be inserted bed-side similar to intrauterine device (IUD) and vaginal ring that are used for contraception, albeit with channels for high dose rate brachytherapy. The device could potentially make cervical brachytherapy easier to the patient, and MRI/CT imaging for treatment planning much more feasible, as the level of discomfort of women with the device inserted is expected to be lower than the current standard of care.

## Intrauterine device for cervical brachytherapy

### Tandem

**Fig. 1:**
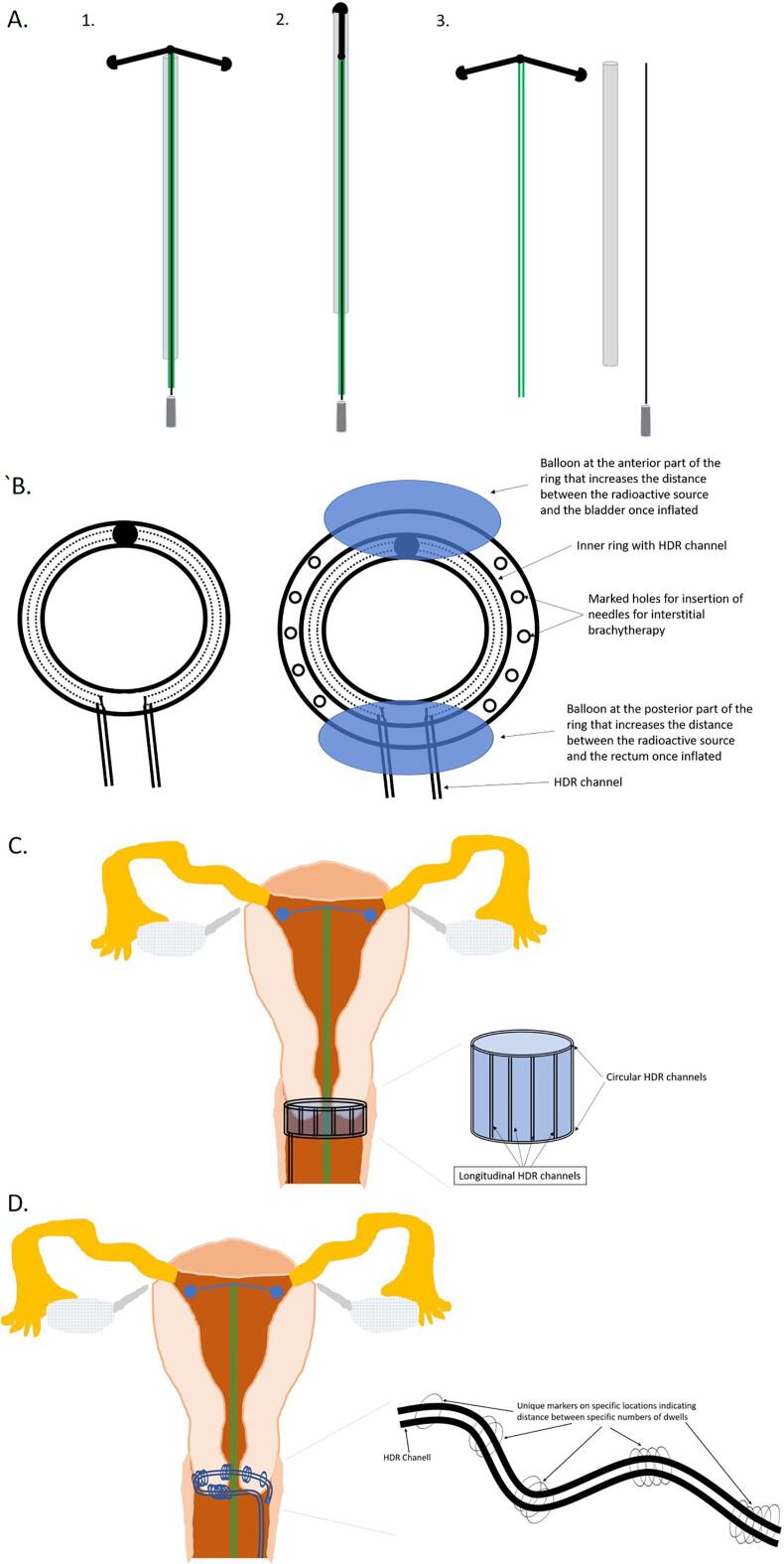
Schematic drawing of the cervical applicator: **A.** Intrauterine HDR Device 1. Baseline condition, 2. Wings housed in the inserter before insertion, 3. Cervical applicator after putting the inserter and guidewire aside. **B.** Cervical ring, with right and left HDR channels, without (left) or with balloons and interstitial brachytherapy holes (right). The balloons are intended to increase the distance between the radioactive source, and the bladder and rectum. **C.** A sleeve shaped applicator with proximal and distal rings, with HDR channels in them, as well as HDR channels vertical to the rings, and parallel to the cervix, within the sleeve. **D.** Another iteration for the ring part of the HDR applicator. A channel that is deposited around the cervix and near the parametria, that has multiple circles, wherein there is a fixed distance between increasing number of circles. The circles are visible under 2D ×-ray, CT, and MRI imaging, and allows determination of the direction of the channel, and the location of the dwells in relation to the tumor, for HDR brachytherapy planning.

**Fig. 2:**
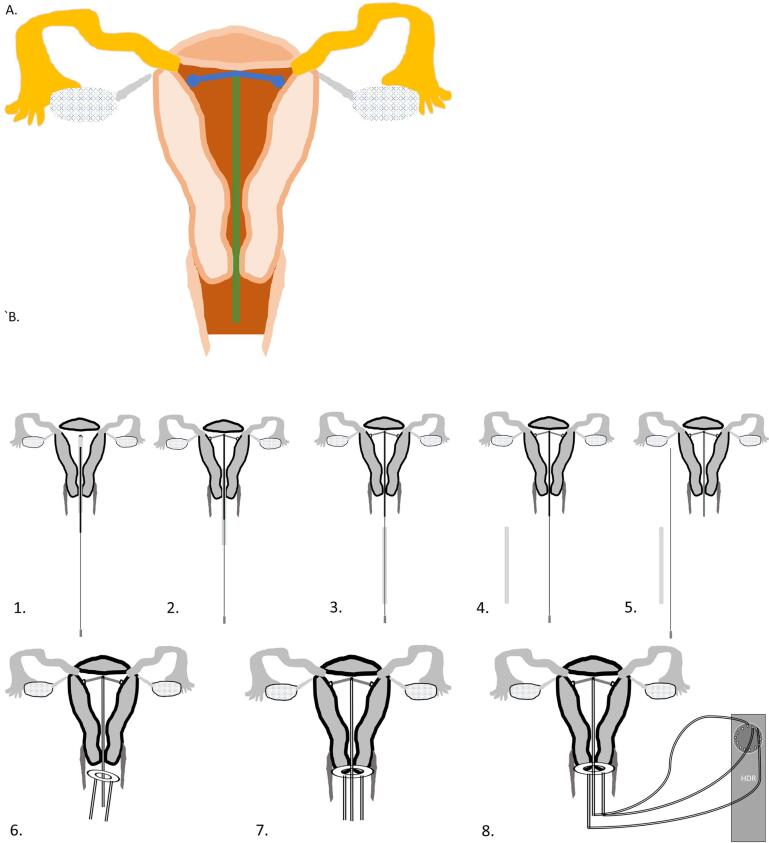
A. The tandem part of the cervical applicator. B. 1. after sounding the uterus, and bringing the wings inside the inserter, the inserter is introduced into the uterus, 2. the inserter is then retracted, allowing the extension of the wings, 3. the inserter is removed while securing the tandem in place by holding the guidewire, 4. then removing the inserter totally, 5. followed by removing the guidewire, 6. the cervical ring is then introduced, 7. and once tandem and ring are in place, imaging is performed, and a plan is generated, 8. the tandem and ring are then connected to the HDR afterloader.

The tandem part of the intrauterine device for cervical brachytherapy (Fig. 1A), is composed of a flexible HDR channel, that connects in its end to two wing shaped flexible arms. The channel has a guide wire in it, so it can be inserted without bending. Before insertion, the uterus is sounded to measure its depth. The inserter is advanced to house the wings (Fig. 1A, 2), which is important in order to make the diameter of the device at time of insertion, identical to that of the inserter. The inserter with the tandem channel in it, is then introduced into the uterus (Fig. 2B, 1). After that, the inserter is removed (Fig. 1A, 3, and Fig. 2B, 2–4), allowing the wings to open, and to anchor the applicator to the uterus. Then, the guide wire is removed (Fig. 2B, 5).

The tandem part could potentially stay through the treatment length of few weeks, so no need for the reinsertion of the tandem at each fraction as is done now days. The part that is out of the cervix will likely be at least 4 cm in length, flexible at its exit from the cervical os, and rigid at its distal 2–3 cm, to allow attaching it to the HDR afterloader. We are investigating designs for the part of the tandem that is out of the cervix, in which it can be long enough to reach outside of the vagina during treatment, to allow easier attachment to the afterloader, and have the distal end flexible to allow bending it, and housing it in the vagina between treatments. The diameter of the applicator part extending out of the cervix will be 2 mm or less, just enough to have the (1 9 2)Ir HDR source, which has a diameter of 0.6 mm [10], to pass into the cervix and uterus. This as opposed to the 4–6 mm rigid outer part used in modern brachytherapy [11].

### Ring

After insertion of the tandem channel, the ring part of the applicator is introduced. The ring is composed of an external stretchable part, that allow it to be “dressed” on the most distal part of the cervix. The ring has two internal channels, one on the right and the other on the left side of the ring, that are attached in their distal end internally at the far most part of the ring, about half a circle away from their entrance point (Fig. 1B). These internal channels are non-stretchable, and thus the distance from the beginning of the channel to its end is fixed (Fig. 1B). The ring can include two inflatable balloons, one between the bladder and the cervix, and the other between the cervix and the rectum (Fig. 1B, right drawing). The role of these balloons is to increase the distance between the cervix and urinary bladder anteriorly, and the between the cervix and the rectum posteriorly. For patients who need interstitial brachytherapy, the ring can include multiple holes to allow insertion of brachytherapy needle channels as needed (Fig. 1B, right drawing).

After insertion of the ring (Fig. 2B, 6–7), MRI/CT scan is performed, and the target volumes and organs at risk are drawn. A brachytherapy plan is generated and the tandem channel as well as the ring channels are then connected to the HDR afterloader for delivery of the treatment (Fig. 2B, 8). After completion of the initial fractions, the ring is removed, but the tandem stay in its place, and is removed only after the final fraction of brachytherapy is delivered.

### Sleeve-like applicator

The ring (Fig. 1B), will be advanced superior to the cervical os, as this will be needed to anchor it, as opposed to the current standard now, in which the ring is close to the cervical os. One solution to this is a sleeve-like applicator, with proximal and distal rings with HDR channels in them, as well as vertical HDR channels in the sleeve (Fig. 1C). The sleeve will be dressed on the most distal part of the cervix, with a number of dwell positions sufficient to achieve dosimetric coverage of the cervical tumor with radiation.

### Flexible longitudinal applicator

For patients of whom the ring cannot fit well, another iteration is a flexible longitudinal applicator composed of one channel that is deposited around the cervix. The flexible longitudinal applicator, has specific markers at fixed distances, such as an increasing number of circles, that allows determination of the direction of the channel, and the localization of the dwells relative to the tumor, for HDR brachytherapy planning (Fig. 1D). The circles will be from radiopaque material for CT planning and from materials hyperintense on T2 for MRI planning. Once this applicator is positioned in its desired location, packing of the vagina with wet pads is done to secure the applicator in place, and to increase the gap between the applicator and the bladder and rectum. Planning is performed taking into account the location of the different parts of the applicator in relation to the tumor, based on the different markers’ locations.

## Discussion and conclusions

Brachytherapy for cervical cancer when combined with EBRT and chemotherapy is highly effective for treating locally advanced disease [3], [4]. The proposed cervical applicator could make the process of brachytherapy technically easier to the physician, less painful to the patient, and likely less costly as there is no need for operation room and general or spinal anesthesia for most patients. Insertion of the tandem part, could potentially be done similar to insertion of IUD without or with local anesthesia only, with a main difference that the tumor at the cervix could make the cervical os less visible and the insertion more challenging. The inserter diameter can be 4.4 mm, similar to that of the Mirena® IUD, 4.0 mm as with Paragard®, or 3.8 mm as with Kyleena® and Skyla® IUDs [12]. The transverse diameter of Mirena® and Paragard® IUDs is 32 mm, and that of Kyleena® and Skyla® is 28 mm [12], so the different sizes of the current invention could be tailored to uterine size and parity status as with the IUDs in market. The outer diameter of some of the commercially available brachytherapy channels is 6F (2 mm), and that can easily fit in either inserter. The HDR source diameter is less than 1 mm [10], and thus it’s a matter of time to develop thinner channels and applicators. The main advantage of the current invention is that the tandem self-anchors to the uterus, and there is no need for Smit sleeve or sutures. The MRI/CT-compatible Venezia applicator (Elekta Brachytherapy, Veenendaal, The Netherlands) has a tandem with an outer diameter of 4 mm and an inner diameter of approximately 2.5 mm [11] but it does not self-anchor to the cervix. The concept that the ring can be fitted on the cervix, rather than affixed to the tandem is new and need to be tested and validated.

The treatment sessions with this invention could be much easier to the patient, as the tandem channel is already in the uterus, as opposed to the current standard of care in which the physician inserts the tandem into the uterus each treatment day. This could save the patient pain and discomfort, and may shorten the procedure time.

Another potential benefit of the current invention is that at the end of the insertion of the tandem IUD, the patient most likely will be able to mobilize and perform normal daily activities, as opposed to the current standard of care in which the patient needs to be in bed all the time the tandem and ovoids are inserted [13]. The patients could potentially be easily mobilized to the MRI or CT suites, and thus volumetric planning could become feasible in every medical center that have these imaging modalities. After planning is complete, the patient is transferred to the HDR treating room, connected to the HDR afterloader and got treated.

Ultrasound based planning for cervical cancer is an emerging technique [14], and the current applicator can potentially be incorporated with this technology by utilizing markers on the applicator visible under US.

The current invention can be used with the Nasser-Zelefsky bladder filling device, that we recently described [15]. This could result in consistently full urinary bladder, through the process of imaging, planning a treatment.

The main drawback of the current invention is that the channel of the IUD will remain in the patients’ vagina for few weeks until completion of treatment. The diameter of the channel is about 2 mm, and is much thicker than the strings used as part of the standard contraception IUDs. Some of the patients will still need cervical dilatation before insertion, as happens with 1–20% of the patients who undergo insertion of contraception IUD [16]. This is likely to be more frequent in patients with cervical tumors due to distorted anatomy. Expulsion of IUDs used for contraception occurs in about 2% of the women at 1 year [17]. The brachytherapy applicator will likely be needed for about a month, and thus the expulsion rates are expected to be less than 2%.

The main challenge is to develop the device and bring it to market with a reasonable price. The current prices of contraception IUDs can reach more than $1000 per unit. The current device will likely save operation room time and anesthesia costs, and will tempt the medical device company to which we will license the invention to try to charge high cost per unit. Cervical cancer is one of the main malignancies in Africa, and my goal as a physician and inventor is to make this device available there at the lowest possible cost.

## Declaration of Competing Interest

The author (N.J.N.) declare the following financial interests/personal relationships which may be considered as potential competing interests: N.J.N. is the inventor on a patent application filed by the University of Maryland Baltimore about the cervical applicator described in the manuscript. N.J.N. is a co-founder of The Umbilicus Inc., a nonprofit organization for preserving sexual function of individuals with cancer below the umbilicus.
